# Facilitation of imitative movement in patients with chronic hemiplegia triggered by illusory ownership

**DOI:** 10.1038/s41598-023-43410-5

**Published:** 2023-09-26

**Authors:** Naoki Aizu, Tamami Sudo, Yutaka Oouchida, Shin-Ichi Izumi

**Affiliations:** 1 Department of Physical Medicine and Rehabilitation, Tohoku University Graduate School of Medicine, 2-1 Seiryo-machi, Aoba-ku, Sendai, Miyagi 980-8575 Japan; 2 Faculty of Rehabilitation, School of Health Sciences, Fujita Health University, Toyoake, Japan; 3 Department of Computer and Information Science, Tokyo University of Agriculture and Technology, Tokyo, Japan; 4 Collective Intelligence Research Laboratory, Graduate School of Arts and Sciences, The University of Tokyo, Tokyo, Japan; 5 Department of Education, Osaka Kyoiku University, Osaka, Japan; 6 Department of Physical Medicine and Rehabilitation, Tohoku University Graduate School of Biomedical Engineering, Sendai, Japan

**Keywords:** Neuroscience, Psychology, Medical research

## Abstract

The sense of body ownership, the feeling that one’s body belongs to oneself, is a crucial subjective conscious experience of one’s body. Recent methodological advances regarding crossmodal illusions have provided novel insights into how multisensory interactions shape human perception and cognition, underpinning conscious experience, particularly alteration of body ownership. Moreover, in post-stroke rehabilitation, encouraging the use of the paretic limb in daily life is considered vital, as a settled sense of ownership and attentional engagement toward the paralyzed body part may promote increased frequency of its use and prevent learned nonuse. Therefore, in addition to traditional methods, novel interventions using neurorehabilitation techniques that induce self-body recognition are needed. This study investigated whether the illusory experience of a patient’s ownership alterations of their paretic hand facilitates the enhancement in the range of motion of succeeding imitation movements. An experiment combining a modified version of the rubber hand illusion with imitation training was conducted with chronic hemiplegia. A larger imitation movement of the paretic hand was observed in the illusion-induced condition, indicating that the feeling of ownership toward the observed limb promotes the induction of intrinsic potential for motor performance. This training, using subjective experience, may help develop new post-stroke rehabilitation interventions.

## Introduction

In recent years, cognitive neuroscience research has indicated that subjective conscious experiences of one’s body are critical. This kind of feeling, known as self-body recognition, involves two basic aspects: a sense of ownership (SoO), described as the conscious experience of an extrinsic body (or part of a body) as one’s own, and a sense of agency (SoA), described as the feeling of controlling movements through one’s own volition^1–3^. Among these subjective experiences, the sense of body ownership is the product of perceived information input integrated with visual, proprioceptive, and somatosensory cues to construct a multisensory representation of the body^4,5^. In general, a hand can be clearly recognized as part of one’s own body if the hand is appropriately positioned to be observed as part of one’s body. This feeling is crucial for accurately perceiving information from the surroundings and performing corresponding adaptive movements. However, SoO can be altered selectively by brain damage, such as stroke, interfering with multisensory integration. In more serious cases, patients may show disturbed sensation of limb ownership^6^, including asomatognosia (unawareness of or ignoring parts or sides of the body) and somatoparaphrenia (a syndrome that includes delusional misidentification and anthropomorphism) that manifest with symptoms such as severe ownership disorder^7,8^. An observational study of stroke patients investigating reduced SoO after brain damage reported that patients with more severe impairment of motor and sensory function presented a greater reduction in body ownership even in cases with no obvious agnosia^9^. Further, in practical clinical cases, patients’ complaints expressing that the hand they are observing is not their own are occasionally reported. The principal sources of these complaints include not only the primary factor such as damage to the areas involved in multisensory integration but also secondary factors such as decreased frequency of use due to impaired motor and sensory functions.

One of the serious problems associated with motor impairment caused by stroke is the further reduction of the frequency of use of the paretic limb by a phenomenon called “learned nonuse,” in which patients learn to use their intact limbs to perform compensatory actions for paretic limbs^10^. When the use of paretic limbs is drastically reduced owing to impairment, the less cortical area is afforded to the paretic limb^11^. However, the brain has the ability to adapt to various changes in the surrounding environment^12–14^; motor training involving frequent use of a specific part of the body, such as constraint-induced movement therapy^15,16^, enlarges cortical representation and accordingly improves motor function^17^. Therefore, even in recent stroke rehabilitation, it is important to provide training to increase the frequency of use of paretic limbs. Research examining the factors associated with the frequency of paretic limb use in stroke patients found that both motor and sensory functions determine the contribution of paretic limbs to activities of daily living^18,19^. Furthermore, studies have highlighted the important role of paretic arm use in daily life with quantitatively evaluated changes in body ownership associated with decreased sensory and motor functions by measuring body-specific attention, which is defined as the amount of attention directed to the body^20,21^. Therefore, for the paretic limb to be used frequently in daily life, merely providing opportunities to use it is not enough. It is necessary to implement a voluntary exercise in a goal-directed action accompanied by recognizing the paretic limb as a part of one’s own body and manipulating it of one’s own volition^22^. For this purpose, in addition to traditional methods, there is a growing need for novel interventions using neurorehabilitation techniques that induce self-body recognition.

Recent methodological advances in research on crossmodal illusions have led to the development of approaches investigating how multisensory interactions shape human perception and cognition, underpinning the conscious experience of one’s own body and its experience^23^. The rubber hand illusion (RHI) is a remarkable phenomenon involving crossmodal input and has been a tool for cognitive neuroscience studies on the perception of visual and somatosensory influences on body representation^24^. In the RHI paradigm, participants watch a rubber hand being stroked while simultaneously being stroked on their real hand, which is hidden from their view. After a period of repeated synchronized strokes, most participants start to feel as if the observed rubber hand is part of their body. Several studies using the RHI paradigm have shown fundamental constraints on eliciting illusory ownership, in which visual and tactile stimulation must be applied at a sufficiently close distance and must be similar in appearance to the participant’s body parts^25–27^. Within these constraints, SoO is more strongly perceived when actual sensory feedback, such as visual, tactile, and proprioceptive information, coincides temporally and spatially with the predicted sensory feedback from these modalities^28^. In some studies, illusion stimuli were presented using a head-mounted display (HMD). Although the virtual body presented through the HMD is different from the body estimated by top-down information, originating from the representation of one’s own body, the experimental settings using HMDs have revealed that the simultaneous visuo-tactile stimulation between actual body parts and an artificial body presented from the first-person visual perspective is sufficient to induce illusory ownership of the artificial body^29–31^. Several imaging studies have also demonstrated that stimulus presentation in an immersive state from the first-person perspective facilitates an appropriate integration of temporally and spatially congruent multisensory signals in a premotor-intraparietal circuit, even in cases of discrepancy between the visual and proprioceptive information about limb position and movement^32–34^. These experimental paradigms have been applied not only in fundamental science research but also in clinical settings to quantitatively describe the mechanisms of bodily self-consciousness and reveal the plasticity of body representation^35–38^.

Recently, imitation training involving frequent use of a specific part of the body with virtual reality technology has been introduced as an example of applying bodily illusion to rehabilitation. This training is based on action observation training^39,40^ that facilitates motor learning in post-stroke rehabilitation by combining physical practice and observation of the same movements, eliciting similar corticomotor representational changes referred to as use-dependent plasticity^41,42^. Furthermore, action observation during imitation activates the neural structures responsible for the execution of these actions in the brain of the observer via a network of neurons known as the mirror neuron system (MNS) and aids in the learning of motor skills^43^. Therefore, we conducted an experiment that combined imitation training with a modified version of the RHI—a virtual hand illusion presented through an HMD. Participants were instructed to perform an imitation movement while illusory ownership was induced. In detail, immediately before they began imitating hand movements, participants’ paretic hands were manipulated by simultaneous visuo-tactile stimulation, inducing illusory ownership in which their observed hands were their own. We then investigated whether the illusory experience of body ownership alterations due to the RHI would facilitate the hand movement of succeeding imitation.

## Results

**Figure 1:**
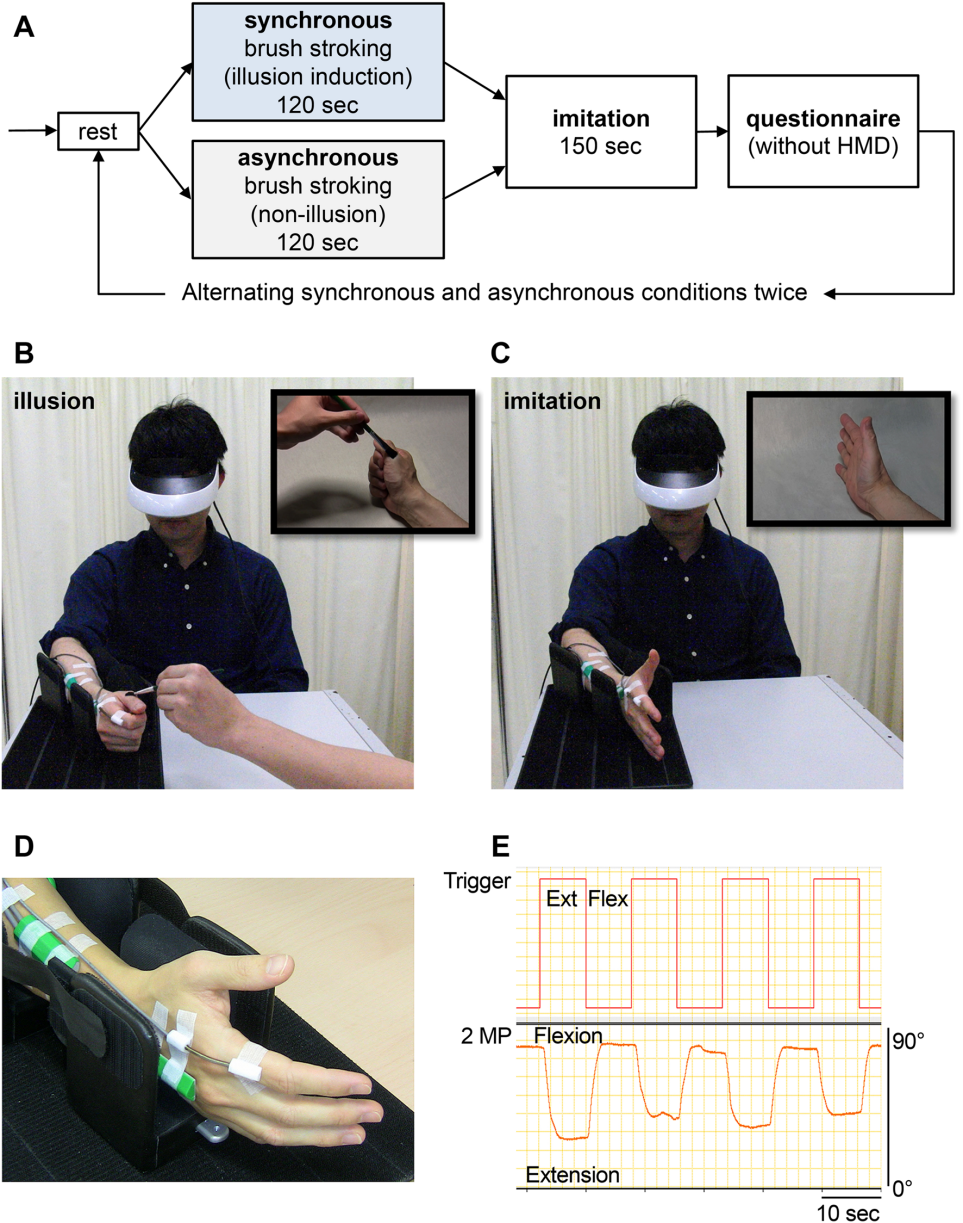
Experimental settings and procedure. (**A**): The experiment consisted of three stages: (1) illusion induction, (2) imitation, and (3) questionnaire. The order of illusions (synchronous and asynchronous) was counterbalanced across participants. (**B**): In illusion induction, the participant’s finger was stroked by a paint brush while they watched a video through the HMD, in which the hand was stroked synchronously or asynchronously. (**C**): In imitation, participants were asked to imitate the cyclic finger open-close movements performed 10 times by the actor’s finger movement in the video stream. The upper right frames in the B and C show cropped images of a part of the video that the participants observing through the HMD. (**D**): An electrical goniometer was attached to the second metacarpophalangeal and wrist joints, and angle changes were recorded during the finger extension and flexion phases. (**E**): Raw data for extension and flexion movements of the second metacarpophalangeal joint.

An experiment combining a modified version of the rubber hand illusion with imitation training was conducted with chronic hemiplegia to investigate whether the illusory experience of a patient’s ownership alterations of their paretic hand facilitates the enhancement in the range of motion (ROM) of succeeding imitation movements (Fig. 1, further details are given under Methods).

**Figure 2:**
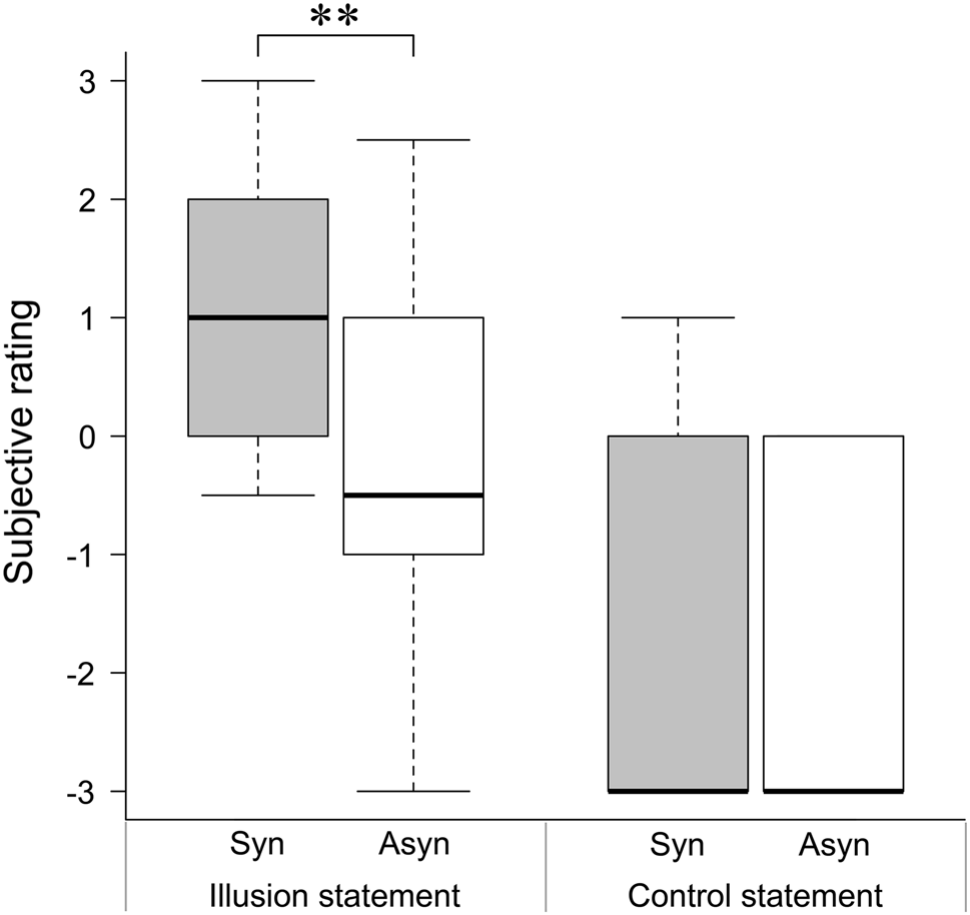
Boxplots for the ratings of questionnaire statements assessing illusory ownership. In the illusion statement, the rating in the synchronous condition was higher than that in the asynchronous condition (*p* = 0.006). However, in the control statement, there were no differences between the ratings in the synchronous and asynchronous conditions. **p < 0.01; Syn, synchronous condition; Asyn, asynchronous condition.

To quantify the subjective experiences of imitation, participants were asked to indicate how much they agreed with the illusion statement, “I felt that the hand I observed in the video stream during the imitation movement was my own paretic hand,” and the control statement, “I felt as if I had more than one paretic hand,” using a 7-point Likert scale ranging from − 3 (I completely disagree) to + 3 (I completely agree). The subjective rating scores for the illusion and control statements are shown in Fig. 2. The main effect of subjective rating was significant, with a higher rating in the synchronous condition than in the asynchronous condition (synchronous condition: median = 1.0; asynchronous condition: median = − 0.5; Wilcoxon signed-rank test: z = − 2.770, *p* = 0.006) for the illusion statement. No such difference was observed for the control statement (synchronous condition: median = − 3; asynchronous condition: median = − 3; Wilcoxon signed-rank test: z = − 0.736, *p* = 0.461).

**Figure 3:**
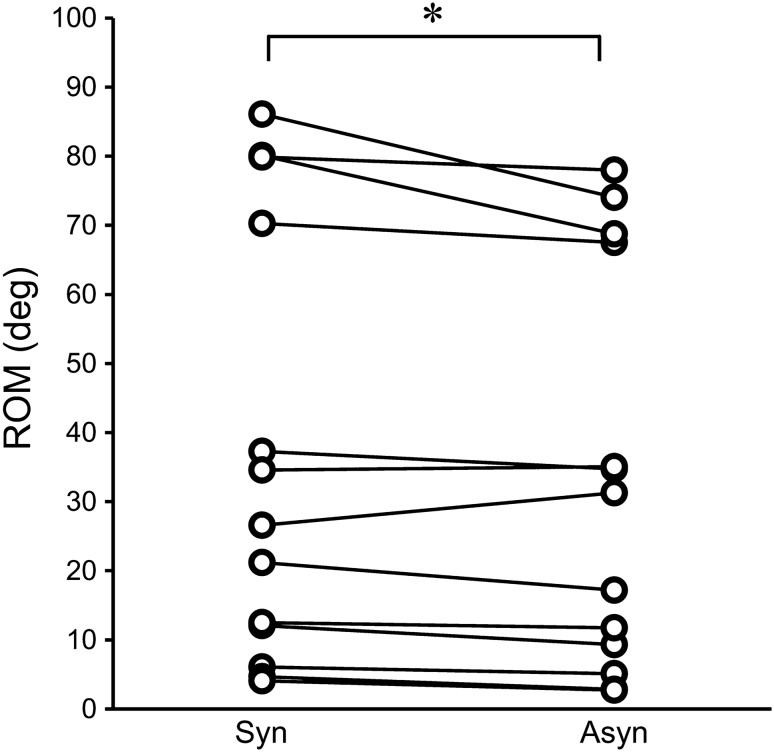
Angular variation of each patient in synchronous and asynchronous conditions. Solid lines indicate data from 13 patients. The angular variation in the synchronous condition was higher than that in the asynchronous condition (*p* = 0.019). *p < 0.05 (Wilcoxon signed-rank test); ROM, range of motion; Syn, synchronous condition; Asyn, asynchronous condition.

Participants were instructed to imitate cyclic finger open-close movements after receiving synchronous or asynchronous visuo-tactile stimulation. The result of comparing the magnitude of ROM measured during the imitation movement presented a significantly greater ROM (36.6° ± 31.5°, Mean ± SD) following synchronous visuo-tactile stimulation, as compared to the ROM (33.7° ± 28.9°, Mean ± SD) following asynchronous stimulation (Wilcoxon signed-rank test: z = − 2.341, *p* = 0.019; Fig. 3). Although the magnitude of ROM varied widely among patients, comparisons of patients among sub-groups did not identify any classifications that reflected variability in the injury side (Mann–Whitney, U = 25.000, *p* = 0.628) or in sensory impairment (Tactile sensation; Mann–Whitney, U = 15.000, *p* = 0.445, Position sense; Mann–Whitney, U = 9.000, *p* = 0.101).

**Figure 4:**
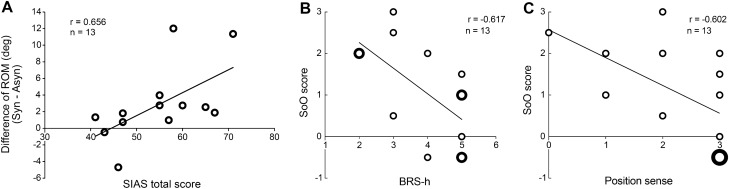
Correlation data. Open circles indicate data plots from 13 patients. The difference in ROM positively correlated with the SIAS total score (**A**). The better the function, the greater the difference between the synchronous and asynchronous finger movements (Spearman’s rank correlation coefficient). The sense of ownership in the synchronous condition was negatively correlated with BRS-h (**B**) and position sense (**C**). The patients have a severe sense of position and severe motor paralysis; hence, they are more likely to feel a strong sense of ownership in the synchronous condition. ROM, range of motion; SIAS, stroke impairment assessment set; Syn, synchronous condition; Asyn, asynchronous condition.

To clarify whether there is a difference in ROM between synchronous and asynchronous conditions depending on the degree of functional impairment of patients, the correlation between the difference in ROM and SIAS total score was examined. The difference in ROM was positively correlated with the SIAS total score (r = 0.656, *p* = 0.015, Fig. 4A). In other words, milder impairment of the patient showed a greater difference between finger movements after synchronous and asynchronous visuo-tactile stimulation, indicating that the effect of synchrony is more pronounced.

Additionally, the subjective evaluation of the illusion statement from the questionnaire was defined as the SoO score, and the relationship between the clinical evaluations and the SoO score for imitation after visuo-tactile stimulation was investigated. The SoO scores after synchronous stimulation were negatively correlated with the BRS-h (r = − 0.617, *p* = 0.025, Fig. 4B) and position sense in the SIAS (r = − 0.602, *p* = 0.029, Fig. 4C). However, the SoO scores after asynchronous stimulation did not correlate with any function. This means that if patients have severe motor paralysis and position sense, they are likely to continue experiencing illusory ownership through synchronous visuo-tactile stimulation.

For a detailed analysis to identify the factors behind a large variability in ratings for the illusion statement in the asynchronous condition, the patients were divided into two groups according to injury side. In comparison by injury side, the patients were divided into the right-brain-damaged group (RBD; N = 7) and the left-brain damaged group (LBD; N = 6). The RBD showed significantly higher ratings for the illusion statement in the asynchronous condition compared to the LBD (Mann–Whitney U = 35.500, *p* = 0.035).

## Discussion

The present study aimed to examine whether patients with chronic hemiplegia can experience illusory ownership by applying crossmodal stimulation, such as RHI, and to investigate the influence of illusory ownership on motor control in imitation. This study has two major findings. First, patients with hemiplegia felt illusory body ownership over the observed actor’s hand in the HMD when the paretic hand was stimulated in the RHI procedure, despite the decrease in the sensory and motor functions of the affected limb. Furthermore, patients with severe paralysis reported stronger illusory ownership. Second, a larger mean ROM of the 2MP joint of the index finger of the patient’s paretic hand was observed in the imitation movement immediately after synchronous visuo-tactile stimulation (in the illusion-induced condition). This result indicates that imitation movements with illusory ownership over the observed hand enhanced the ROM of the paretic hand.

### Induction of bodily illusion to paretic limbs

To monitor the body’s state and configuration, the brain constantly updates its own information in the surrounding environment. This precisely stored spatio-temporal information regarding the current body state in the brain is defined as the body representation^44^. A recent study revealed that the alteration of body representation is strongly associated with the feeling of bodily self-consciousness derived from self-generated action, visual, and tactile sensation from the surrounding environment and proprioception. Among these, self-generated action plays the most important role in strongly inducing bodily self-consciousness^45^. Comparing the actual body state obtained by interacting with the environment by the self-generated action with the predicted body state obtained from the efference copy of the motor command and sensory information generates a prediction error. The long-term absence of self-generated action disrupts the process of updating the prediction error, resulting in the mislocalization of one’s own body parts and interruption in the monitoring of one’s current body state. This condition is also observed in patients with chronic hemiplegia who have distorted body representation with long-term nonuse of the paretic limbs according to their own will. Many hemiplegic patients continue to lack sufficient sensory feedback from the external environment through spontaneous movements that are important for monitoring their current body state. Thus, patients tend to compensate for inaccurate monitoring, owing to the absence of movement by using visual information to understand their body state^36^. As a result, affected limbs with reduced functional and sensory function after brain injury are more susceptible to the illusory effect^37,38,46^. In this study, most patients reported a strong sense of ownership of the actor’s hand in the video stream. In particular, the results of the correlation between the SoO scores and the severity of patient injury (Fig. 4B, C) indicate that the more severe the paralysis, the stronger the illusion, which is consistent with previous clinical studies^36–38,46^.

The unusual integration strategy brought about by such dysfunction also causes excessive reliance on visual information that supplements somatosensory and proprioceptive information, leading to the localization of one’s body parts being more obscure, resulting in a decrease in attention to the self^20^, stronger RHI due to less reliable proprioception^47^ and a decrease in the sense of ownership of their own body^9^. Recent psychophysical experiments have shown the opposite effect of suppressing tactile detection and promoting visual detection after RHI^48^. Other studies have investigated the amount of attention directed to the self-body by a quantitative index called body-specific attention; more visual attention was directed to the limbs with lower tactile abilities and post-amputation prostheses, compensating for the lack of sensory information^49,50^. These characteristics suggest a tradeoff between sensory and visual feedback. Thus, in the process of multisensory integration under the bodily illusion, visual feedback of the position and state of the body existing in space plays a dominant role over proprioceptive and somatosensory feedback. The simultaneous visuo-tactile stimulation in the present experiment was superposed from the first-person perspective to provide an immersive feeling. The visual-dominant sensory integration observed in this experimental setting allowed to induce an illusory ownership even in severely paralyzed patients who could not sufficiently move their paretic hand and could not achieve the same movement as the one presented in the HMD. The difference between synchronous and asynchronous ROM is greater in patients with milder disability, indicating that the effect of the illusion during the imitation phase is more pronounced (Fig. 4A). Whereas in patients with more severe paralysis, the effect of the illusion in the imitation phase is certainly existence but small. The slight effect in these severe patients may be attributed to the discrepancy between visual and actual movements due to the inability to perform the same movement presented in the video. Moreover, such sensory integration depends on visual feedback and is prominent in patients with right hemisphere lesions^51^. Patients belonging to the RBD group in this study also experienced the illusory ownership regardless of visuo-tactile simultaneity. This result reflects the characteristics of sensory integration in patients with hemiplegia, specifically those with right hemisphere lesions, as described by Martinaud et al.

### Predominance of visual feedback in multisensory integration

The predominance of visual feedback in crossmodal integration also lies in the crucial process in the human motor system, minimizing the prediction error and preparing for the subsequent motor output based on the sensory feedback of the previous state. This mechanism has a strong influence on motor control in subsequent movements with illusory changes in ownership. Post-illusory changes caused by visual dominance were observed, even in experiments with healthy blindfolded subjects. In other modified RHI paradigms (i.e., illusory self-touch) with no visual clues of the position of one’s hands within space due to blindfolds, the brain must depend only on proprioceptive and tactile information as a clue to figure out an accurate representation of their own hand. As a result, the localization of touch becomes less accurate, leading to an enhanced illusory effect^52–54^. Other behavioral studies employing the reaching task after RHI induction in healthy individuals demonstrated that illusory ownership by RHI manipulation updates body schema regarding the starting position, posture, and trajectory for appropriate hand movements based on visual feedback^55,56^.

In another novel RHI paradigm, including unexpected movements of the fake hand during illusion induction, participants’ spontaneous hand movements were observed to correspond to the movement of the fake hand. Moreover, EEG analyses in this study revealed greater mu-rhythm desynchronization of the motor system during observation of fake hand movements with a strong feeling of body ownership over the fake hand^57^. Studies that measured motor-evoked potentials demonstrated the possibility of inhibitory effects on corticospinal pathways, triggered by a short-term illusory perception of not being ready to use the body part during observation of a fake hand with missing or movement restriction^58,59^. On the other hand, contrary to these reports, a recent study revealed that manipulating body ownership with RHI in healthy participants has little impact on motor cortical excitability^60^. Collectively, these findings indicate that the induction of the illusion in the present experiment is not necessarily an intervention that entails the risk of reducing the patient's motor output in actual body. This evidence suggests that illusory ownership of the observed movement determines the output of one’s voluntary movement based on visual feedback, resulting in the emergence of brain activity, corresponding to actual self-execution. In addition to the static RHI, which provides visuo-tactile stimulation in a non-moving state, the moving RHI, in which a fake hand moves simultaneously, has also been reported^61,62^. In this illusion with movement, owing to spatio-temporal matching between visual information and proprioceptive feedback received from the body parts in action, the sense of ownership of the observed fake hand is preserved even during the action. A recent neuroimaging study during the moving RHI revealed that there was a cortical site associated with both agency and body ownership and combined their information. Furthermore, there was an interaction of neural representation between body ownership and agency in the somatosensory cortex, suggesting an agency-induced ownership enhancement of somatosensory cortical activity specific for voluntary movement^63^. The findings of these previous studies suggest that the patients in this study might have reported continuous experience of the illusory effects during imitation, despite the illusion-induced stimulation with the visuo-tactile brush stroking being presented only in the first half of the video presentation. The experimental setting of this study played a role in inducing ideal movement while presenting the movement of the mobilized hand and providing an immersive feeling with the HMD. It is considered that this setting led to the imitation movement accompanied by a significant change in the finger angle after the synchronous visuo-tactile stimulation, despite the difficulty of movement by the paretic hand. Such imitation training is also expected to promote the transformation of the neural plasticity of body representations at the cortical level and is effective in post-stroke rehabilitation.

### Facilitation effect of paretic limb movement by MNS

The second interpretation of the significantly larger patients’ finger movement by illusory ownership of the observed hand movement is that the MNS is involved in the back projection of perceived movement. During imitation, specific brain areas that are engaged in both action observation and action execution are activated. This matching activation is a function of a network called an MNS^64,65^. These MNS-based mechanisms are related to cognitive functions, such as imitation learning and motor memory formation, which can be strengthened through observation of congruent action^66,67^. Human imitation involves the flow of information processing across three principal cortical areas: the superior temporal sulcus (STS), inferior parietal lobule (IPL), and inferior frontal gyrus (IFG). The STS provides higher-order visual processing of observed action while the MNS of the frontoparietal region (the IPL and IFG) codes for the goal of the action and the motor plan on how to achieve it. The MNS sends the efferent copies of the motor plan to the STS to check the predicted consequence of the planned action against the visual description of the observed action^68,69^.

Several functional imaging studies revealed that human mirror neurons are selective to actions within the observer’s motor repertoire, and when observing novel actions of others not part of one’s motor repertoire, higher-order visual processing performed in the STS works selectively^70,71^. As indicated by the answers to the questionnaire in the current study, the patients recognized the observed movement as a self-produced movement due to the illusory ownership of the hand observed through the HMD. Thus, our results suggest that patients temporarily judged the observed movement as an action included in their own motor repertoire by the bodily illusion presented through the HMD. In addition, recorded fMRI data from expert dancers have revealed that the areas of MNS showed greater activation when observing the specific movements that they are accustomed to performing than when observing movements that they are familiar with but not accustomed to executing^72^. Based on these findings, it is suggested that imparting body ownership to a virtual body presented from a first-person perspective using an HMD enhances MNS activity and induces an efficient motor output to reproduce the target movement.

### Limitations and future research directions

This study had several limitations. First, considering the physical condition of the patients, only a few types of question statements were prepared. Specifically, including statements confirming whether SoA changed through imitation was necessary. However, since such statements were not part of the current procedure, the implication of SoA could not be discussed in this study. Since there was no movement in the RHI in the first half of the procedure, it is presumed that the change in SoO was purely induced, whereas the later imitation may have caused the amplification or attenuation of SoO. The questionnaire evaluations of pre- and post-imitation may have been able to distinguish between the effects of the first and last half of the procedure. Second, individual differences depended on the lesion site and were uncontrollable. Difference of illusory effects was found depending on the injury side, but this grouping was not originally planned. Thus, the sample size was insufficient by group for a detailed discussion; hence, studies with more participants for each injury side are required. Third, the effects shown in this study were revealed by comparing the conditions with and without the illusion. Thus, how the effects of general rehabilitation training itself were involved remains unclear. Finally, the current study is only a preliminary verification that leads to a temporary improvement of imitative movement in patients with hemiplegia. To apply such a procedure to rehabilitation, further investigation is needed to confirm whether continuous intervention translates the immediate effect into a long-term effect.

In this study, we suggest that the feeling of ownership toward an observed limb is conducive to perceptual changes in bodily self-attribution and to the induction of intrinsic potential for motor performance. To consider this phenomenon as enhanced motor output, a paradigm that correlates it with motor performance in various types of patients should be constructed, including healthy subjects, using various types of indicators such as grip strength, electromyographic pattern, reaching velocity, and reaching distance. Our findings are also instructive in that the concept of bodily illusion, which has generally been examined within fundamental science, provides insight into rehabilitation medicine. This type of training, which utilizes an immersive subjective experience to enhance SoO, may improve the frequency of use of paretic limbs and increase training opportunities with self-body recognition. These conclusions may contribute to innovations in effective post-stroke rehabilitation strategies.

## Methods

### Participants

A total of 13 patients with chronic hemiplegia resulting from supratentorial lesion (hemorrhage: 7, infarction: 5, trauma: 1) participated in this study. The sample size had been pre-determined prior to the experiment by considering effect size (0.8), power (0.8), and significance level (0.05) using G*Power soft-ware. The estimated number of participants for this study is broadly consistent with that of previous studies on RHI for hemiplegic patients^37,46^. The inclusion criteria were as follows: participants should (1) be at least 18 years of age, (2) have at least six months post-brain damage, and (3) demonstrate cognitive and communication skills sufficient to understand study instructions. The exclusion criteria were: (1) flaccid paralysis; (2) complete tactile loss (to administer the RHI); (3) unilateral spatial neglect; (4) hemianopia; (5) global aphasia; and (6) other serious, uncontrolled medical or psychological conditions. The mean age of the participants was 56.2 ± 12.1 years. All participants had some motor paresis and sensory disorders (see Table 1 for details of patients’ symptoms).

**Table 1:** Clinical characteristics of patients.

No	Age	Gender	Cause	Paretic side	Lesion location	Duration since onset	SIAS			
							Finger function test	Tactile sensation	Position sense	Total score
1	60	M	I	R	P, IC, CR	27	1A	3	3	57
2	68	M	I	R	P, CR	51	1A	3	3	51
3	52	M	I	R	P, IC, CR, Pa, CN	58	3	1	1	67
4	62	M	I	L	P, IC, CR, Pa, CN	47	1A	2	2	55
5	60	F	I	L	P, CR	35	2	3	3	55
6	41	M	H	R	P, T, CR	32	1A	2	2	41
7	64	M	H	R	CR	10	1C	3	3	43
8	67	F	H	R	P, IC, CR, T	50	1B	2	2	47
9	49	M	H	L	P, IC, CR	54	2	3	3	65
10	46	M	H	L	CR	7	4	3	3	71
11	66	M	H	L	P, IC, T	36	3	2	1	46
12	67	M	H	L	P, IC, CR, T	192	1A	1	0	47
13	29	M	T	L	DAI	117	4	3	3	60
		11 M/2F	5I/7H/1 T	6R/7L						
Median	60					47	1C	2	3	55
Range	29–68					7–192	1A-4	1–3	0–3	41–71

M, male; F, female; I, infarction; H, hemorrhage; T, trauma; R, right; L, left; P, putamen; IC, internal capsule; CR, corona radiata; T, thalamus; Pa, pallidum; CN, caudate nucleus; DAI, diffuse axonal injury. In the finger function test, a score of 1A means they can perform synkinetic flexion of fingers; 1B, synkinetic extension of fingers; 1C, partial separative flexion or extension of fingers; 2, incompletion of finger movement; 3–4, completion of finger movement with clumsiness; 5, normal status; and 0, total paralysis. In tactile sensation and position sense, a score of 0 means loss; 1, severe; 2, mild; and 3, normal.

Prior to commencing the experiments, several assessments were carried out, including the Stroke Impairment Assessment Set (SIAS) for motor and sensory functions^73,74^ and the Brunnstrom Recovery Stage (BRS)^75^ for the severity of motor paralysis. The SIAS can evaluate the multifaceted functional impairment of stroke, including the motor function (0–5 points) and sensory function (0–3 points), for a total score of up to 76 points. A motor function score of 0 indicates complete paralysis and corresponds to the exclusion criteria in this study (flaccid paralysis). Subitems of the motor function included the finger function test (0–5 points), and those of the sensory function included tactile sensation and position sense (0–3 points each). To investigate the correlation with the experimental data, besides the total score, subitems of the finger function test, tactile sensation, and position sense were extracted and added to the analysis. The BRS represents the severity of paralysis on a 6-point scale, from 1 (flaccid paralysis) to 6 (normal function returns). In addition, two conventional spatial neglect screening tests, the horizontal line-bisection task^73,74^ and cancellation task^76^, were conducted to confirm the absence of attentional bias to the visual field.

All participants were recruited from Tohoku University Hospital. The study protocol was approved by the Medical Ethics Committee of Tohoku University School of Medicine (2010–203). The methods were carried out in accordance with approved guidelines, and all participants provided written informed consent before the experiment.

### Experimental setup and procedure

Participants were asked to sit in a chair, rest their arms on a table in front of them, and wear an HMD (iWear VR920 Video Eyewear, Vuzix Corporation; HMZ-T1, Sony Corporation) that played a pre-recorded video stream. In the video stream, the hand of a healthy adult (referred to as the actor’s hand) was shown from a first-person perspective as a substitute for the participant’s paretic hand and was presented to overlay where the participant had placed their paretic hand (Fig. 1).

Each session of this experiment consisted of three stages: (1) illusion induction, (2) imitation, and (3) questionnaire (Fig. 1A). In the first part of the experiment (illusion induction), while playing the first half of the video stream, the experimenter administered one of two kinds of stroking (synchronous or asynchronous) to the participant’s paretic hand for a period of 120 s (48 strokes in each stroking session). In the video, another experimenter’s hand with a paintbrush appeared and randomly started stroking the thumb and the index and middle fingers of the actor’s hand at a speed of about 0.4 Hz. In the synchronous condition, the experimenter, sitting opposite the participant, delivered brushstrokes to the participant’s paretic hand, matching the timing and pattern of the brushstrokes administered to the corresponding locations on the actor’s hand in the video stream (Fig. 1B). In the asynchronous condition, the stimulation administered to the participant’s paretic hand was temporally and spatially incongruent with that delivered to the actor’s hand in the video stream. Next, participants were instructed to imitate the actor’s finger movement while observing the latter half of the video stream (Fig. 1C). The video contained 10 periodic cyclic finger open-close movements performed slowly over approximately 150 s by the actor’s hand, which had previously been stroked by a brush. The finger movement in the video stream was performed at a sufficiently low speed to allow all the patients to achieve the cyclic imitation movement. Since switching between flexion and extension was not easy depending on patient severity, triggers by click sounds were inserted into the video sequence every 7.7 s to guide the timing of the finger extension and flexion movements.

During imitation, the amount of flexion or extension angle in the participants’ movements was sequentially monitored using an electrical goniometer (Biometrics Ltd, F35). The device was placed on the second metacarpophalangeal (2MP) joint of the paretic index finger to evaluate the effect of each condition (Fig. 1D). To eliminate factors of angular variation of the finger caused by wrist movement, the paretic upper limb was fixed in a forearm-neutral position with the wrist joint slightly extended. Another goniometer (Biometrics Ltd., SG65) was attached to the wrist joint to monitor the wrist without movement. After the imitation, the participants were assessed for the magnitude of illusory ownership of imitation using modified questionnaires from a previous report^23^. The HMD was removed when the questionnaire was provided to the participants. An approximately 3-min rest was allowed before the start of the next session when the patient put on the HMD again. In this experiment, a total of four sessions, constructed as an ABAB or BABA design, were conducted, including two conditions for each synchronous and asynchronous stroking.

### Questionnaire: subjective measurement of the effect of the illusion

To quantify the subjective experiences of imitation, participants were asked to indicate how much they agreed with the illusion statement, “I felt that the hand I observed in the video stream during the imitation movement was my own paretic hand,” and the control statement, “I felt as if I had more than one paretic hand,” using a 7-point Likert scale ranging from − 3 (I completely disagree) to + 3 (I completely agree). The illusion statement and the control statement were used to evaluate the key perceptual component of the illusion and as a control for task compliance, respectively. Thus, a total of four questionnaire results were collected, two each after imitation movement in the synchronous and asynchronous conditions.

### Flexion and extension angles: objective behavioral measurement of training

The recorded angle data from the 2MP joint of the index finger of the patient’s paretic hand were amplified and recorded using an analog-to-digital converter (Power Lab 16/35; ADINSTRUMENTS, Japan) and an analysis software (Lab Chart 7; ADINSTRUMENTS). The maximum extension and flexion angles were obtained from each of the extension and flexion phases, indicated by waveforms corresponding to visual stimuli (Fig. 1E). The amplitude of the angular variation, calculated as the difference between the maximum extension and flexion angles of each phase, was defined as the range of motion (ROM). In each session, 20 ROMs were acquired 10 times each from the extension and flexion phases, and the average value was calculated and analyzed.

### Statistical analysis

The Shapiro–Wilk test was used to determine the distribution of the datasets. For normally distributed datasets, a paired t-test was applied to analyze the difference in the means between the two conditions. For datasets that were not normally distributed, the Wilcoxon signed-rank test was applied. In addition to the data obtained from the experiment, such as the difference in ROM and the subjective evaluation from the questionnaire, the clinical scores of the SIAS (total score, finger function test, tactile sensation, and position sense) and the Brunnstrom Recovery Stage of hand (BRS-h; the severity of finger paralysis) were analyzed.

The correlation between the difference in ROM (obtained by subtracting the value in the asynchronous condition from the value in the synchronous condition) and the SIAS total score was examined to determine whether the difference in ROM between synchronous and asynchronous conditions depends on the functional impairment of patients. The correlation between the subjective evaluation from the questionnaire and motor and sensory functions of the patient’s hand (such as the BRS-h and subitems in the SIAS) was examined using Spearman’s rank correlation coefficient to investigate the factors that affected changes in SoO during imitation.

## Data Availability

The datasets generated during and/or analyzed during the current study are available from the corresponding author on reasonable request.
